# Genomic analysis of oesophageal carcinoma (EC) identifies recurrent mutations in histone methyltransferases as a distinctive subset

**DOI:** 10.1038/s41388-026-03897-4

**Published:** 2026-07-11

**Authors:** Jingyuan Wang, Joanne Xiu, Francesca Battaglin, Hiroyuki Arai, Shivani Soni, Wu Zhang, Richard M. Goldberg, Anthony F. Shields, Axel Grothey, Jimmy J. Hwang, John L. Marshall, Igor Astaturov, Benjamin A. Weinberg, Emil Lou, Michael Hall, Rachna T. Shroff, Moh’d Khushman, Mohamed E. Salem, Davendra P. S. Sohal, Aaron J. Scott, Matt Oberley, Tianshu Liu, Lin Shen, Heinz-Josef Lenz

**Affiliations:** 1 Department of Medical Oncology, Zhongshan Hospital, Fudan University, Shanghai, China; 2 Department of Medicine, Norris Comprehensive Cancer Center, Keck School of Medicine, University of Southern California, Los Angeles, CA USA; 3 Department of Oncology, Zhongshan Hospital (Xiamen), Clinical Research Center for Precision Medicine of Abdominal Tumor of Fujian Province, Fudan University, Shanghai, China; 4 Caris Life Sciences, Phoenix, AZ USA; 5 West Virginia University Cancer Institute, Morgantown, WV USA; 6 Department of Oncology, Karmanos Cancer Institute, Wayne State University, Detroit, MI USA; 7 West Cancer Center and Research Institute, Germantown, TN USA; 8 Levine Cancer Institute, Carolinas HealthCare System, Charlotte, NC USA; 9 Ruesch Center for The Cure of Gastrointestinal Cancers, Lombardi Comprehensive Cancer Center, Georgetown University Medical Center, Washington, DC USA; 10 Fox Chase Cancer Center, Philadelphia, PA USA; 11 University of Minnesota, Minneapolis, MN USA; 12 Department of Clinical Genetics, Fox Chase Cancer Center, Philadelphia, PA USA; 13 Division of Hematology/Oncology, University of Arizona Cancer Center, Tucson, AZ USA; 14 Medical Oncology, Washington University in St. Louis/Siteman Cancer Center, St. Louis, MO USA; 15 University of Cincinnati, Cincinnati, OH USA; 16 Division of Hematology and Oncology, Banner University of Arizona Cancer Center, Tucson, AZ USA; 17 Key laboratory of Carcinogenesis and Translational Research (Ministry of Education/Beijing), Department of Gastrointestinal Oncology, Peking University Cancer Hospital and Institute, Beijing, China

**Keywords:** Predictive markers, Prognostic markers

## Abstract

Histone-lysine N-methyltransferase 2 (KMT2) family proteins methylate lysine 4 on the histone H3 tail at important regulatory regions in the genome and thereby impart crucial functions through modulating chromatin structures and DNA accessibility. We aimed to identify the molecular profile of KMT2-MT oesophageal cancer (EC) and associations with patient outcomes. In our study, *KMT2* mutations were significantly associated with longer immunotherapy-related overall survival (OS) in the esophageal adenocarcinoma (EA) (21.42 vs 13.42 months, HR = 0.66, 95% CI: 0.50–0.88, *P* = 0.004), but not in the esophageal squamous cell carcinoma (ESCC). *KMT2* mutations were significantly associated with microsatellite instability-high and higher tumor mutation burden in both EA and ESCC. *KMT2*-MT EA showed significantly higher mutation rates in *CIC* (13% vs 1.1%), *NF1* (12.5% vs 2.2%), *FBXW7* (12% vs 3.4%), *ATM* (11.5% vs 2.6%) and *BRCA1* (11.5% vs 1%, all q < 0.05), compared to *KMT2*-wildtype (WT) tumors. Meanwhile, no significant mutational differences were observed between *KMT2*-MT and WT ESCC. Fat digestion and absorption, cholesterol metabolism and the infiltration of activated B cells were significantly enriched in *KMT2*-MT EA compared to *KMT2*-WT EA, but these results were not observed in the ESCC. This is the largest study to investigate the distinct molecular landscapes in KMT2-MT EC, characterized by higher tumor mutational burden, an increased frequency of microsatellite instability-high, and gene mutations involved in DNA damage repair and epigenetic regulation.

## plain language summary

Histone-lysine N-methyltransferase 2 (KMT2) family showed a frequent mutation rate in oesophageal cancer, which may have an impact on patient outcomes. Clinical and genetic information from patients with oesophageal adenocarcinoma (EA) and oesophageal squamous cell carcinoma (ESCC) was compared between the KMT2 mutant and wild-type subgroups. We found that KMT2 mutations were associated with survival benefit with immunotherapy in EA, but this association was not clearly seen in ESCC. A unique molecular profile in EA patients with *KMT2* mutations is defined by higher rates of concomitant mutations in the DNA damage repair pathway, which might predict sensitivity to PARPi and immunotherapy.

## Introduction

Epigenetic changes that lead to alterations in the chromatin structure and the accessibility of transcriptional machinery to DNA, but are not associated with changes in the DNA sequence, are indispensable in tumorigenesis [1]. Epigenetic dysregulation in cancer cells may lead to the activation of oncogenes or repression of tumor-suppressor genes, driving cancer initiation and progression. Histone modification is one of the crucial regulatory processes of epigenetic mechanisms that target arginine and lysine residues of H3 and H4 histones, which can regulate gene expression and maintain chromatin structure at the corresponding sites [2, 3]. Deregulation of histone H3 on lysine 4 methylation (H3K4me) is commonly detected across a wide range of human cancers and has been suggested to influence the tumor immune environment [4]. KMT2A, a member of the histone-lysine N-methyltransferase 2 (KMT2) family, has been reported to increase programmed cell death ligand 1 (PD-L1) expression by enhancing H3K4me3 levels in the promoter regions of CD274, which may further promote immune escape in both esophageal squamous cell carcinoma (ESCC) and pancreatic carcinomas [5, 6]. KMT2D loss was reported to increase DNA damage and mutation burden, promote chromatin remodeling and IFN-γ-stimulated antigen presentation via the decreased levels of H3K4me1 [7].

Esophageal cancer (EC) is the seventh most common cancer globally [8]. The introduction of immune checkpoint inhibitors (ICIs) targeting PD-1/PD-L1 has reshaped the landscape of cancer therapy across many types of tumors, including EC [both ESCC and esophageal adenocarcinoma (EA)]. Conclusions from the ATTRACTION-3 and KEYNOTE-181 studies suggested that anti-PD-1 inhibitors have emerged as a novel option in the second-line treatment for patients with metastatic EC [9, 10]. Moving forward, in the first-line treatment of metastatic EC, anti-PD-1 inhibitors in combination with chemotherapy/anti-CTLA4 agents have also demonstrated similarly promising results in KEYNOTE-590 and Checkmate-648 [11, 12]. Despite the improvement observed, some patients still do not experience benefits from the inclusion of ICI treatment. The use of biomarkers to guide ICI treatment in EC is clinically required, and this requirement was recently endorsed by the Food and Drug Administration’s (FDA) Oncologic Drug Advisory Committee (ODAC).

Genomic studies in EC have identified frequent mutations in histone methylation, such as KMT2 family, indicating that aberrations in epigenetic machinery play vital roles in the initiation and progression of EC [13]. However, a comprehensive analysis of molecular features and the immune phenotype of EC patients with *KMT2* mutations has not yet been fully characterized. Our study was designed to explore the efficacy of ICIs and the distinct molecular features in *KMT2*-mutant (MT) EC based on a large database established by a commercial CLIA-certified laboratory (Caris Life Sciences, Phoenix, USA) (referred to as CARIS).

## Material and methods

### Patients and tumor samples

This study examined a total of 787 patients, including 604 EA and 183 ESCC, from the CARIS database, whose tumor sample analyses were anonymously paired with clinical records. Genomic, transcriptomic and clinical data of TCGA were publicly downloaded from cbioportal (http://www.cbioportal.org/).

### Targeted next-generation sequencing

Tumor enrichment was achieved by harvesting targeted tissue from formalin-fixed paraffin-embedded (FFPE) tumor samples using manual microdissection techniques. NGS and gene variant calling were carried out using the NextSeq for 592 whole-gene targets (Agilent Technologies, Santa Clara, CA) or NovaSeq 6000 for more than 700 clinically relevant genes (>700x) and an additional >20,000 genes (>200x) (Illumina, Inc., San Diego, CA) [14]. All variants were detected with > 99% confidence based on allele frequency and amplicon coverage, with an average sequencing depth of coverage of > 500 and an analytic sensitivity of 5%. comparing this calculated result with a precalibrated value. Genetic variants identified were interpreted by board-certified molecular geneticists and categorized as “pathogenic,” “presumed pathogenic,” “variant of unknown significance,” “presumed benign,” or “benign,” according to the American College of Medical Genetics and Genomics (ACMG) standards. When assessing mutation frequencies of individual genes, “pathogenic,” and “presumed pathogenic” were counted as mutations while “benign”, “presumed benign” variants and “variants of unknown significance” were excluded. Tumor mutation burden (TMB) was measured by counting all non-synonymous missense, nonsense, in-frame insertion/deletion and frameshift mutations found per tumor that had not been previously described as germline alterations in dbSNP151, Genome Aggregation Database (gnomAD) databases, or benign variants identified by Caris geneticists. Caris Life Sciences is a participant in the Friends of Cancer Research TMB Harmonization Project.

### Immunohistochemistry (IHC)

Using automated staining techniques, IHC staining was performed on slides obtained from FFPE tumor blocks as previously described (Benchmark XT; Ventana; and AutostainerLink 48; Dako, Carpinteria, CA) [15]. PD-L1 (SP142) was stained, and positivity was defined as ≥2 + , >5% staining on tumor cells.

### Microsatellite instability-high (MSI-H)

MSI-H status of the tumors profiled using MSI fragment analysis (Promega, Madison, WI) and NGS (for tumors tested with the NextSeq platform, 7000 target microsatellite loci were examined and compared to the reference genome hg19 from the University of California).

### Statistical analysis

Statistical analysis was performed with R (version 4.0). The comparisons of continuous data were analyzed using the Mann-Whitney U test to reflect a difference in the overall distribution. The comparisons of categorical data were analyzed using the Fisher-Exact or Chi-square test. The Benjamini-Hochberg test was used to adjust for multiple co-mutation comparisons. The single-sample gene set enrichment analysis (ssGSEA) was used to compare the abundance of immune cells between *KMT2*-MT and wild-type (WT) EC samples. To analyze and visualize the results, we employed the “ggplot2” package for correlation analysis. The comparison of gene mutation rates and gene expression between the *KMT2*-MT and *KMT2*-WT groups of the TCGA cohort was analyzed using the “DESeq2” R package and was enriched by Kyoto Encyclopedia of Genes and Genomes (KEGG) analysis. For survival analysis, Kaplan-Meier curves were compared using the log-rank test. Immune-related overall survival (irOS) was defined as the time from initial immunotherapy (nivolumab or pembrolizumab treatment) to the day of death. A two-sided *p* < 0.05 was considered statistically significant.

## Results

### Study population and KMT2 status

**Fig. 1:**
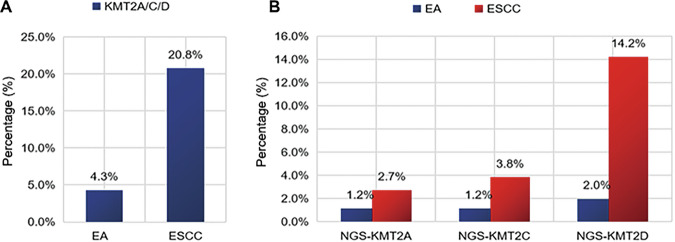
The mutation status of KMT2 in EC. **A** KMT2 mutation in EA and ESCC, respectively. **B** KMT2A, KMT2C, and KMT2D mutations in EA and ESCC, respectively.

Due to the distinct biological features between EA and ESCC, we analyzed *KMT2* mutations in the EA and ESCC cohorts separately. Overall, *KMT2* mutations were more frequently observed in ESCC than in EA (20.8% versus 4.3%) (Fig. 1A). In ESCC, 5 (2.7%), 7 (3.8%) and 26 (14.2%) of patients showed *KMT2A*, *KMT2C* and KMT2D mutations, respectively. Meanwhile, 7 (1.2%), 7 (1.2%) and 12 (2.0%) of EA patients showed *KMT2A*, *KMT2C* and *KMT2D* mutations, respectively. In both cohorts, *KMT2D* was the most commonly mutated among the KMT2 family genes (Fig. 1B).

### Treatment and outcome analysis

**Fig. 2:**
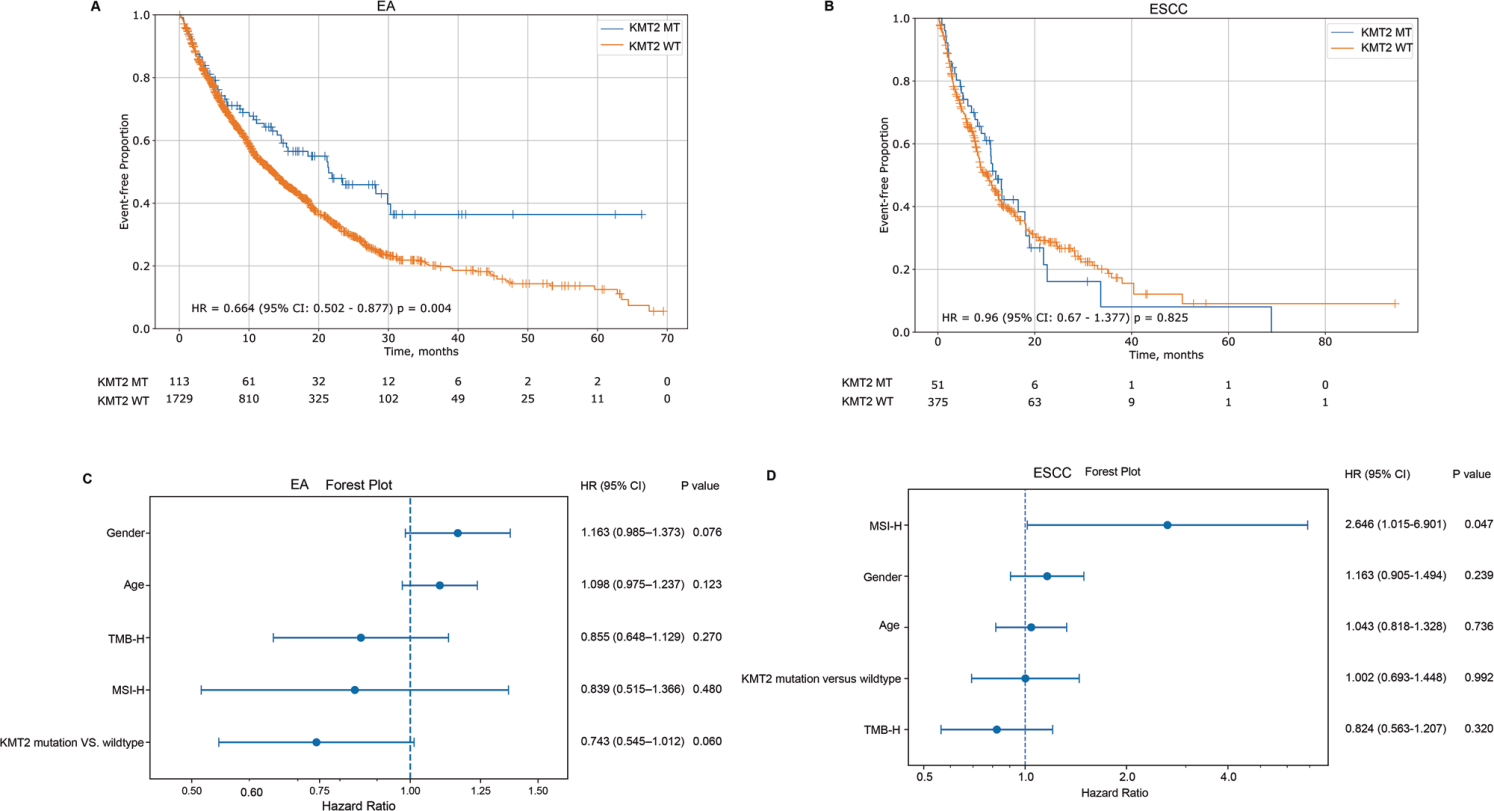
The impact of KMT2 mutations on the survival of EC patients receiving ICI treatment. **A** KMT2 mutation was positively associated with the clinical efficacy of patients treated with ICIs in EA. **B** No significant difference in the association of KMT2 mutations with the efficacy of ICI treatment was observed in ESCC. The multivariate COX analyses were conducted to explore the impact of KMT2 mutations on the survival of EA (**C**) and ESCC (**D**) patients receiving ICI treatment.

EA patients whose tumors exhibited *KMT2*-MT had a significantly longer irOS, compared to those with *KMT2*-WT tumors (21.42 vs 13.42 months, HR = 0.66, 95% CI: 0.50–0.88, *P* = 0.004, Fig. 2A). This association was also observed in the multivariable Cox analysis (HR 0.74, 95% CI 0.54–1.01, *P* = 0.06, Fig. 2C). No significant difference in irOS was identified between ESCC patients with KMT2-MT and KMT2-WT tumors, both in univariate and multivariable analyses (median OS: 11.94 months vs. 10.20 months, univariate HR = 0.96, 95% CI 0.67–1.38, *P* = 0.825, Fig. 2B; multivariable HR = 1.0, 95% CI 0.69–1.45, *P* = 0.99, Fig. 2D).

### KMT2 mutations are associated with MSI-H and higher TMB

**Fig. 3:**
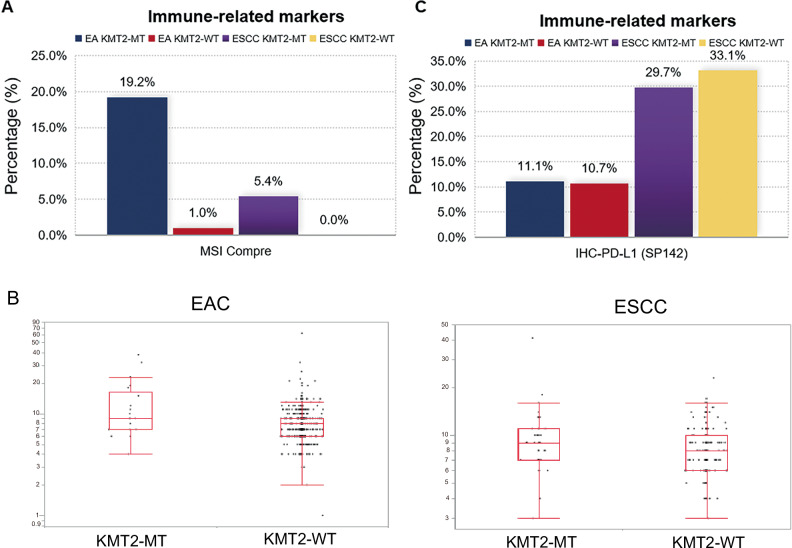
The association between *KMT2* mutations and predictive biomarkers for response to ICI treatment. **A** The association between *KMT2* mutations and MIS-H. **B** The association between *KMT2* mutations and TMB. **C** The association between *KMT2* mutations and PD-L1 expression.

Next, we analyzed biomarkers associated with a predictive value for response to ICI treatment. *KMT2* mutations were strongly associated with MSI-H (EA: 19.2% vs 1.0%; ESCC: 5.4% vs 0%, Fig. 3A) and higher TMB (median 9 vs 8 MT/Mb, Fig. 3B) in both EA (*P* < 0.0001) and ESCC (*P* = 0.042) cohorts. Even after excluding MSI-H samples, the association of *KMT2* mutations with higher TMB remained significant in the EA (*P* = 0.008) and ESCC (*P* = 0.015) cohorts. There was no significant association with PD-L1 expression between *KMT2*-MT and WT subgroups in both EA and ESCC cohorts (Fig. 3C).

### Alterations in the *KMT2*-MT cohort, compared to the *KMT2*-WT cohort

**Fig. 4:**
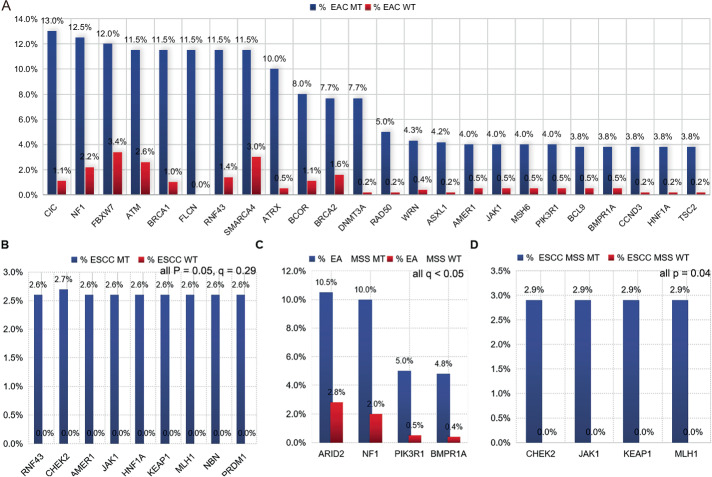
Genomic features of *KMT2*-MT EC, compared to *KMT2*-WT EC. **A** Genomic features of KMT2-MT EA, compared to KMT2-WT EA. **B** Genomic features of KMT2-MT ESCC, compared to KMT2-WT ESCC. **C** Genomic features of KMT2-MT EA, compared to KMT2-WT EA in the MSS subgroups. **D** Genomic features of KMT2-MT ESCC, compared to KMT2-WT ESCC in the MSS subgroups.

*KMT2*-MT EA was associated with a higher rate of co-incident mutations in *CIC* (13.0% vs 1.1%), *NF1* (12.5% vs 2.2%), *ATM* (11.5% vs 2.6%), *BRCA1* (11.5% vs 1.0%), *FLCN* (11.5% vs 0%), *RNF43* (11.5% vs 1.4%), *ATRX* (10.0% vs 0.5%), *BCOR* (8.0% vs 1.1%), *DNMT3A* (7.7% vs 0.2%), *RAD50* (5.0% vs 0.2%), *WRN* (4.3% vs 0.4%), *ASXL1* (4.2% vs 0.2%), *CCND3* (3.8% vs 0.2%), *HNF1A* (3.8% vs 0.2%) and *TSC2* (3.8% vs 0.2%), compared with *KMT2*-WT EA (all *q* < 0.05, Fig. 4A).

However, mutations in *RNF43* (2.6% vs 0%), *CHEK2* (2.7% vs 0%), *AMER1* (2.6% vs 0%), *JAK1* (2.6% vs 0%), *HNF1A* (2.6% vs 0%), *KEAP1* (2.6% vs 0%), *MLH1* (2.6% vs 0%), *NBN* (2.6% vs 0%) and *PRDM1* (2.6% vs 0%) occurred more frequently in *KMT2*-MT ESCC, although the association did not reach statistical significance (all *P* = 0.05, *q* = 0.29, Fig. 4B).

Because MSI-H status may trigger secondary mutations, we performed a subgroup analysis in microsatellite-stable (MSS) cases. A higher mutation rates of *NF1* (10% vs 2%), *PIK3R1* (5% vs 0.5%) and *BMPR1A* (4.8% vs 0.4%) remained significant in the *KMT2*-MT EA cohort, compared with the *KMT2*-WT EA cohort (all *q* < 0.05, Fig. 4C). Meanwhile, in the ESCC cohort, there were more *CHEK2* (2.9% vs 0%), *JAK1* (2.9% vs 0%), *KEAP1*(2.9% vs 0%) and *MLH1* (2.9% vs 0%) mutations in the *KMT2*-MT subgroups than those in the *KMT2*-WT subgroups (all *p* = 0.04, Fig. 4D).

### Transcriptomic features in the *KMT2*- MT cohort, compared to the *KMT2*-WT cohort

**Fig. 5:**
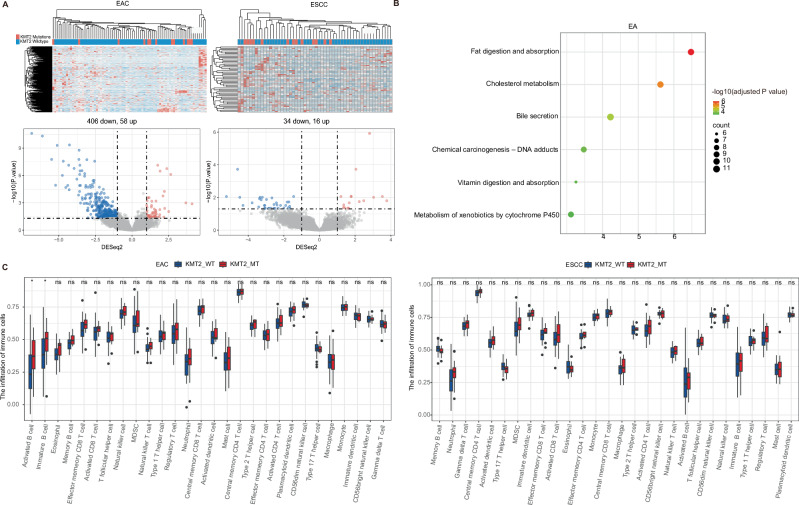
Transcriptomic features of *KMT2*-MT EC, compared to *KMT2*-WT EC. **A** DEGs were compared between *KMT2*-MT and WT tumor samples in EA and ESCC, respectively. **B** KEGG analysis for DEGs in the EA cohort. **C** The infiltration of immune cells was compared between *KMT2*-MT and WT tumor samples in EA and ESCC, respectively.

To understand how *KMT2* mutations influence the gene expression pattern in EA and ESCC, respectively, we performed a comparison of mRNA expression between the *KMT2*-MT and WT patients using the TCGA database. A total of 464 differentially expressed genes (DEGs) were identified between *KMT2*-MT and WT EA patients, most of which were enriched in fat digestion and absorption and cholesterol metabolism (Fig. 5A, B). The infiltration of activated B cells was significantly higher in *KMT2*-MT EA patients (Fig. 5C)

However, only 50 DEGs were found by the comparison between *KMT2*-MT and WT ESCC patients, and no signaling pathway was enriched for these genes (Fig. 5A). No significant difference in the infiltration of immune cells was observed between *KMT2*-MT and WT ESCC patients (Fig. 5C).

## Discussion

Members of the KMT2 family methylate histone H3 on lysine 4 (H3K4), promoting genome accessibility and transcription, leading to the increase of TMB and the production of neoantigens [7]. A transcriptome-wide association study (TWAS) revealed that the KMT2 gene family was not associated with the risk of EC in both UTMOST and FUSION analyses (Table S1–2), which suggested that mutations in the KMT2 family may probably be concomitant events, rather than driven events during the pathogenesis of EC, consistent with previous reports [16]. Our study reports for the first time the impact of *KMT2* mutations on the survival of patients receiving ICI treatment and the molecular characteristics in both EA and ESCC.

ESCC and EA were reported to have unique characteristics of mutational profiles and the tumor immune environment [17]. EA has been shown to demonstrate higher frequencies of mutations in *APC*, *ARID1A*, *KRAS*, and *SMAD4*, as well as HER2 overexpression. ESCC demonstrated higher PD-L1 expression compared to EA [17]. Our study showed that *KMT2* mutation rates were higher in the ESCC than in the EA cohorts.

*KMT2* mutations have been reported to be associated with survival benefit with ICI treatment in melanoma, uterine cancer, non-small cell lung cancer (NSCLC) and colorectal cancer [18]. In our studies, we found that *KMT2* mutations were only related to longer OS in EA, but not in ESCC. Further exploration found that the differences of MSI-H, TMB, mutational and transcriptomic features between *KMT2*-MT and WT tumors were more significant in EA than those in ESCC. In EA, most of the gene mutations co-occurring with *KMT2* mutations, such as *ATM*, *BRCA1*, *SMARCA4*, *ATRX*, *BRCA2*, *RAD50* and *WRN*, were related to DNA damage repair (DDR) pathways. Chang et al. revealed that KMT2C regulates DDR through direct recruitment to DNA damage sites by Ago2 and small non-coding RNA, where it mediates H3K4 methylation, chromatin relaxation, secondary recruitment of DDR factors, and amplification of DDR signals along chromatin in NSCLC [19]. Loss of KMT2D could induce ROS-mediated DNA damage by suppressing the enhancer activity and DNA binding of antioxidant transcription factor FOXO3 in prostate cancer [20]. Hereby, *KMT2C/D* mutations may sensitize cancer cells to Poly(ADP-ribose) polymerase inhibitors (PARPi). In consideration of enrichment for MSI-H and TMB-high tumors in KMT2-MT EA, a multivariate COX analysis has confirmed the association of survival benefit with KMT2 mutations in EA. Besides, the infiltration of activated B cells and gene expression levels associated with metabolic processes involved in fat digestion and absorption, as well as cholesterol metabolism, were higher in *KMT2*-MT EA than those in *KMT2*-WT EA. In pancreatic cancer, the inactivation of KMT2D promotes tumour growth and results in loss of the H3K4me3 mark, which further activates the expression of SLC2A3, leading to increased FA synthesis [21]. It has been shown that fatty acids can enhance the capacity of dendritic cells (DCs) to exhibit increased in vitro and in vivo chemotaxis, accompanied by better stimulatory and cytotoxic T cell activity as well as a favorable T helper cell 1 (Th1) cytokine profile [22]. Activated B cells, represented by plasma cells and memory B cells, could provide T cells with stronger signals, such as the inducible costimulator (ICOS) ligands CD80 and CD86, to support the function of T cells in immune killing [23]. B-cell activation by T follicular helper cells and the secretion of antibodies is a key reflection of the response to immunotherapy in breast and colorectal cancer [23]. Wang et al. revealed that Kmt2d-mutant cells exhibited increased IFNγ-stimulated antigen presentation and immune infiltration [7]. However, no significant differences in gene mutations and enriched pathways for DEGs between *KMT2*-MT and WT subgroups were observed in ESCC. The limited number of ESCC tumors may reduce statistical power. In addition, the significance of the association with higher TMB was more remarkable in EA than in ESCC. These results might provide the theoretical basis for the improved clinical benefit for *KMT2*-MT subgroups in EA, but not in ESCC.

Limitations of this work need to be mentioned. First, the retrospective nature of our study may introduce selection bias; thus, prospective randomized studies with a larger sample size of EA and ESCC integrating genome-wide genotype, transcriptomic, and immunotherapy outcome data are warranted to further confirm the predictive value of KMT2 mutations for ICI treatment. Second, clinical information regarding the line of therapy and combination regimens was not available. Third, the functional roles of *KMT2* mutations in EC should be further explored in vitro and in vivo to explore the impact of mutations in the KMT2 family on metabolic processes and the tumor immune environment.

## Conclusions

In summary, this is the largest comprehensive molecular characterization of *KMT2*-MT EC. Our data suggest a unique molecular profile in EA patients with *KMT2* mutations defined by higher rates of concomitant mutations in the DDR pathway, which might predict sensitivity to PARPi and ICI treatment. Further clinical trials are needed to assess the efficacy of these therapies in *KMT2*-MT EA patients.

## Supplementary information

Table S1. The risk of EC in the FUSION analysis

Table S2. The risk of EC in the UTMOST analysis.

## Data Availability

The study protocol and statistical analysis plan are available in the paper. Other data (including the summary of clinical and genomic data) will be made available upon reasonable request. (jingyuanwang1992@gmail.com, jxiu@carisls.com).
